# Emerging Treatments for Obesity: the Role of GLP1 Receptor Agonists on Stroke

**DOI:** 10.1007/s11910-025-01423-9

**Published:** 2025-05-21

**Authors:** Melissa Mariscal, Fernando D. Testai

**Affiliations:** Neurology and Rehabilitation, University of Illinois Chicago, 912 S Wood St, Chicago, IL 60612 USA

**Keywords:** Stroke, Obesity, GLP-1, GLP1RAs, Cardiovascular disease

## Abstract

**Purpose:**

Stroke is a leading cause of disability and mortality worldwide. It has been estimated that more than 90% of the risk of stroke is associated with modifiable factors, including diabetes, hypertension, obesity, and heart disease. Glucagon–like peptide 1 receptor agonists (GLP1RAs) have been shown to have a beneficial effect on these major risk factors. In this review, we discuss the evidence supporting the use of GLP1RAs on brain health, particularly in relation to stroke prevention.

**Recent Findings:**

The results of multiple randomized clinical trials demonstrate that, among patients with type 2 diabetes, GLP1RAs reduce body weight and improve glucose levels and lipid metabolism. In high-risk populations, GLP1RAs also reduce the risk of major adverse cardiovascular events, including all stroke and non-fatal stroke. Mechanistically, GLP1RAs have a beneficial effect on different stroke risk factors, support microvascular function, and reduce inflammation and oxidative stress.

**Summary:**

GLP1RAs are recommended for the primary prevention of stroke in patients with diabetes and elevated cardiovascular risk.

## Introduction

Glucagon–like peptide 1 receptor agonists (GLP1RAs) are a cutting-edge anorectic therapy option with growing popularity. By reducing appetite, delaying gastric emptying, and enhancing the secretion of insulin, these drugs reduce body weight, improve glucose levels, and enhance lipid metabolism. The evidence obtained in multiple randomized clinical trials demonstrates that GLP1RAs are effective for the treatment of type 2 diabetes, obesity, and dyslipidemia. There is, in addition, emerging evidence that indicate these new drugs can have cardioprotective and neuroprotective properties. The on-label and off-label use of GLP1RAs has gained popularity, driven by robust clinical research, documented benefits, and widely circulated claims on social media [1]. In addition, professional treatment guidelines recommend the use of these medications in populations at high risk for developing major adverse cardiovascular events. This chapter reviews the history, benefits, and side effects of GLP1RAs use in relation to brain health with special emphasis on stroke.

### History and Pharmacology

**Fig. 1:**
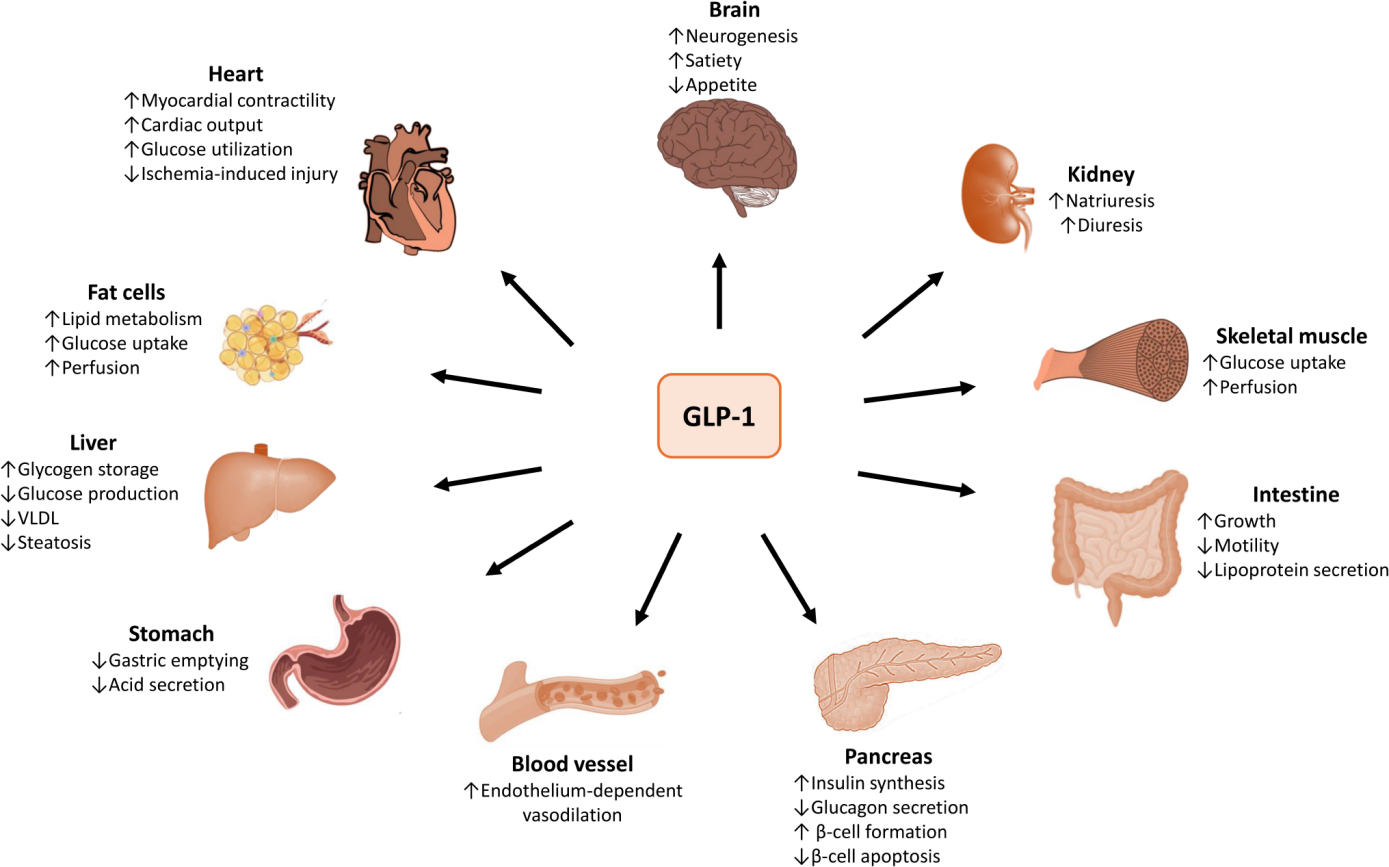
Effect of Glucagon-like peptide 1 (GLP-1). GLP-1 receptors are ubiquitously expressed in the body. In the gastrointestinal system, GLP-1 reduces intestinal motility, gastric emptying, and glucagon secretion. In addition, it increases insulin synthesis and enhances lipid metabolism. In the cardiovascular system, it reduces blood pressure, enhances cardiac function, and protects against ischemia-induced injury. In the central nervous system, it reduces appetite and increases neurogenesis

**Fig. 2:**
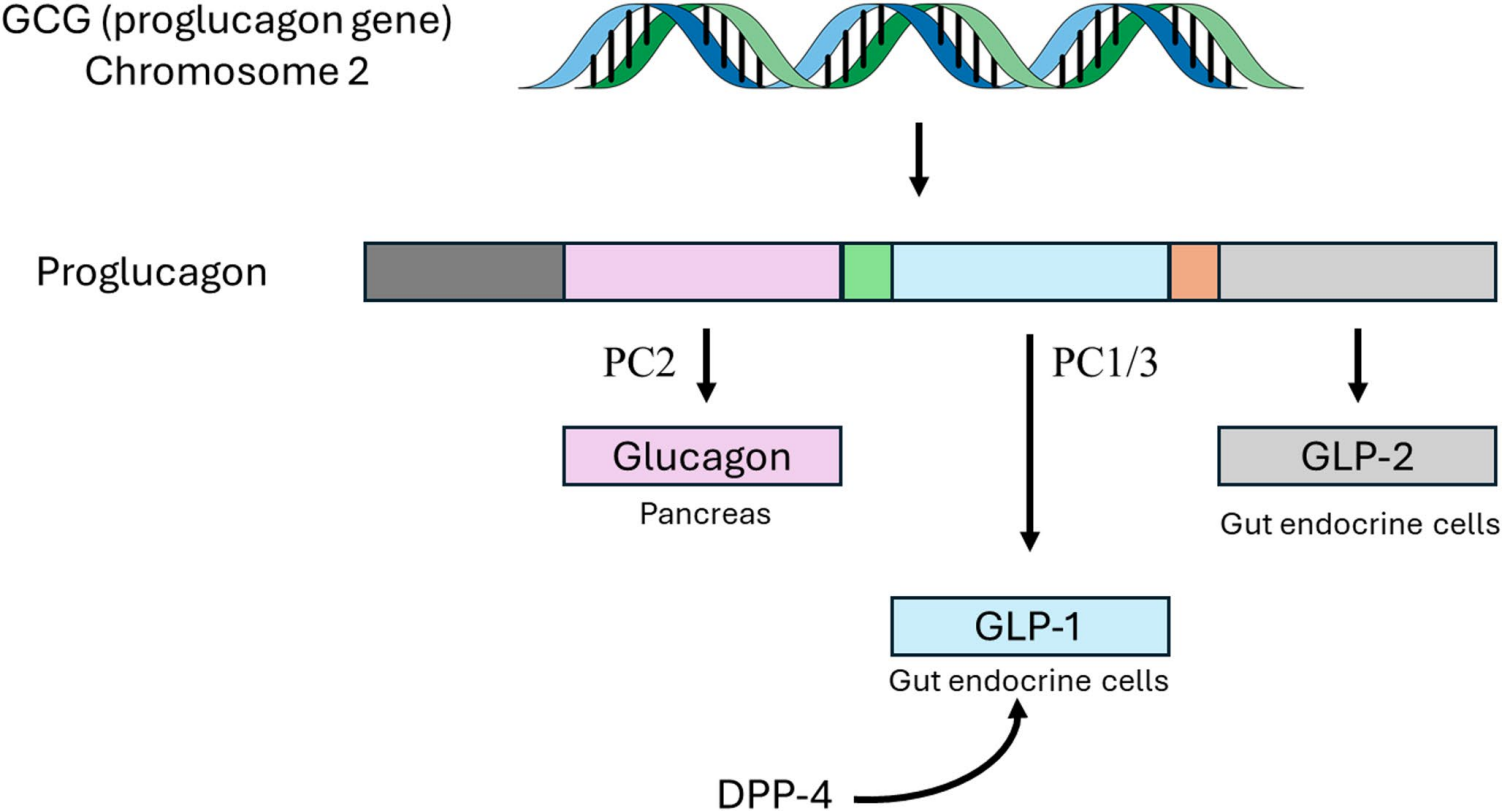
Biochemistry of proglucagon. The GCG gene in chromosome 2 encodes for proglucagon, which has a tissue specific processing. In the pancreas, the enzyme prohormone convertase 2 (PC2) metabolizes proglucagon into glucagon which is stored in vesicles. Hypoglycemia and the glucose-dependent insulinotropic peptide stimulate the secretion of glucagon which increases glycemic levels. In the intestine and the brain, proglucagon is metabolized by the prohormone convertase 1/3 (PC1/3) into glucagon-like peptide 1 (GLP-1) and glucagon-like peptide 2 (GLP-2). GLP-1 suppresses appetite, inhibits the secretion of glucagon, and stimulates the release of insulin, all processes that reduce the circulating levels of glucose and body weight. GLP-2, in comparison, enhances intestinal epithelial barrier function and reduces intestinal inflammation. Glucagon, GLP-1, and GLP-2 are degraded by dipeptidyl peptidase 4 (DPP-4).

In the 1970s, the glucose-dependent insulinotropic polypeptide (GIP) was isolated from porcine gastric extracts [2]. The discovery of GIP, a potent stimulator of insulin secretion, laid the foundation for the successful mapping of the proglucagon gene and the discovery of the glucagon-like polypeptides GLP-1 and GLP-2. GLP-1 and GIP are incretins, hormones produced by the epithelium of the gastrointestinal tract in response to food intake that promote the secretion of insulin. GLP-1 is synthesized in different cells, including pancreatic islet cells, intestinal mucosa L-cells, and neurons in the nucleus solitary tract [3]. GLP-2, in comparison, promotes intestinal epithelial proliferation, decreases inflammation and apoptosis, and enhances gut barrier function. By activating its ubiquitously expressed receptor GLP1R, GLP-1 regulates multiple physiological processes which are summarized in Fig. 1. GLP-1 delays gastric emptying and causes postprandial satiety. In addition, it increases the secretion of insulin by the pancreas and plays a pivotal role in glycemic control and lipid metabolism [4]. Thus, GLP-1 was rapidly identified as a potential new treatment for diabetes and metabolic syndrome. Incretins, however, are rapidly degraded by dipeptidyl peptidase-4 resulting in fleeting half-lives of 1–2 min (Fig. 2). The next important step in the development of GLP1RAs occurred in 2005 when exendin-4, a GLP-1-like peptide with a half-life of more than 2 h, was isolated from Gila monster venom. Although exendin-4 still lacked the extended half-life required to be effective for glycemic control, it set the foundation for the development of new GLP-1 analogs relatively resistant to dipeptidyl peptidase-4 with prolonged half-lives revolutionizing the treatment of obesity and type 2 diabetes. Currently, there are two types of GLP1RAs. Exenatide, lixisenaide, and efpeglenide are based on the sequence of exendin-4. In comparison, albiglutide, dulaglutide, and semiglutide are human-GLP-1 based analogs. These therapy options differ by half-lives and modes of administration (Table 1).

**Table 1:** Characteristics of glucagon-like peptide 1 receptor agonists

Drug Name	Type	Mode of Administration	Dosages	Regimen	Therapeutic time frame	Half-life
Exenatide	Synthetic form of Exendin-4 present in Gila monster	Subcutaneousiinjection	5 µg and 10 µg	Twice Daily: Administered twice a day before breakfast and dinner	Short-Acting	~ 2.5 h
Lixisenatide	GLP-1 receptor agonist	Subcutaneous injection	10 µg for 14 days 20 µg thereafter	Daily	Short-Acting	~ 3 h
Liraglutide	Synthetic analogue of human GLP-1	Subcutaneous injection	1.2 mg and 1.8 mg	Daily	Intermediate-Acting	~ 13 h
Abiglutide *discontinued	Long-acting GLP-1 agonist w/ GLP-1 dimer fused with human serum albumin	Subcutaneous injection	30 mg, 50 mg	Once Weekly	Long-Acting	~ 6–7 days
Semaglutide	GLP-1 agonist with high homology to human GLP	Subcutaneous injection	0.25 mg, 0.5 mg, 1 mg, 2 mg	Once Weekly	Long-Acting	~ 7 days
Semaglutide Oral formulation	GLP-1 agonist with high homology to human GLP	Oral	3 mg, 7 mg, 14 mg	Daily	Long-Acting	~ 7 days
Dulaglutide	GLP-1 receptor agonist	Subcutaneous injection	0.75 mg 1.5 mg Max 4.5 mg	Once Weekly	Long-Acting	~ 3.75 days
Tirzepatide	Dual GIP and GLP-1 receptor agonist	Subcutaneous injection	2.5 mg, 5 mg, 7.5 mg, 10 mg, 12.5 mg, 15 mg	Once Weekly	Long-Acting	~ 5 days
Exenatide-ER *discontinued	GLP-1 agonist	Subcutaneous injection	2 mg	Once Weekly	Long-Acting	~ 5 days

### Effect on Obesity and Diabetes

Obesity, particularly central obesity, is a major contributor to metabolic syndrome, which includes insulin resistance, type 2 diabetes, dyslipidemia, and hypertension. In addition, it has been linked to multiple noncommunicable pathologies such as diabetes, cancer, non-alcoholic fatty liver, obstructive sleep apnea, cardiovascular disease (CVD), and stroke [5]. The term obesity epidemic refers to the rapid and widespread increase in the burden of this condition around the globe. World Health Organization estimates show that in 2022 1 in 8 people in the world had obesity, which doubled compared to data from 1990. Also, according to the Center of Disease Control (CDC), approximately 40% of the adults living in the US have obesity (BMI ≥ 30 kg/m^2^) and 9.4% have severe obesity (BMI ≥ 40 kg/m^2^). Despite the implementation of educational campaigns, the prevalence of obesity and severe obesity among children and adolescents in the US increased from 5.2% to 1.0% in 1971–1974 to 19.3% and 6.1% in 2017–2018, respectively. In comparison, the prevalence of obesity in adults increased from approximately 28% in 1999–2000 to 43% in 2017–2018 [6].

Several studies have assessed the role of GLP-1 in obesity. In the randomized double-blind controlled study STEP-1 (Research Study Investigating How Well Semaglutide Works in People Suffering From Overweight or Obesity), the treatment of adults with obesity once-weekly with semaglutide at a dose of 2.4 mg resulted in a mean reduction in body weight of approximately 15%, compared to 3% in the control group [7]. In addition, the coprimary end point of weight reduction of ≥ 5% was achieved in 86% of participants in the group receiving semaglutide and 32% of those randomized to placebo. In the study SCALE (Effect of Liraglutide on Body Weight in Non-diabetic Obese Subjects or Overweight Subjects With Co-morbidities: SCALE™ - Obesity and Pre-diabetes), patients with obesity and pre-diabetes were randomized to once-daily subcutaneous injections of liraglutide at a dose of 3.0 mg or placebo. After 56 weeks of treatment, the mean loss of weight was 8.4 kg in the study group and 2.8 kg in the control group [8]. In a meta-analysis that included 8 randomized clinical trials (RCT) of semaglutide with overweight or obese patients *without diabetes*, the mean weight of participants receiving the study drug was 10 kg lower than in those receiving placebo [9]. Similarly, in another meta-analysis including 47 RCT and more than 23 thousand patients, patients receiving different GLP1RAs had a mean difference in weight of -5 kg relative to placebo [10]. This difference was more pronounced in individuals without diabetes (-9.2 kg) than in those with diabetes (-2.7 kg). Thus far, there are only two FDA-approved medications for weight loss in the GLP1RAs family: semaglutide and tirzepatide (Table 2).

**Table 2:** Major GLP-1RA. Indications for FDA approval, side effects, and contraindications

Drug	Date	FDA approval	Side effects	Contraindications
Liraglutide	2010	Improve glycemic control in adults with type 2 diabetes	Nausea, vomiting, diarrhea	Hypersensitivity reaction History of pancreatitis or develops pancreatitis Family or personal history of Thyroid C-cell tumors/medullary thyroid cancer or multiple endocrine neoplasia 2 (MEN2) Pregnancy
	2014	Chronic weight loss management		
	2017	Pediatric patients with (10 years and older) with type 2 diabetes		
	2023	First generic version approved for type 2 diabetes		
Semaglutide	2017	Type 2 diabetes	Nausea, vomiting, diarrhea	Hypersensitivity reaction History of pancreatitis or develops pancreatitis Family history of medullary thyroid cancer or multiple endocrine neoplasia 2 (MEN2) Pregnancy
	2021	Chronic Weight Management		
	2023	Obesity in 12-year-old and older		
	2024	Reducing risk of cardiovascular events in adults with obesity/overweight and cardiovascular disease		
	2025	Reducing risk of kidney disease progression, kidney failure, and cardiovascular death in adults with type 2 diabetes and chronic kidney disease		
Dulaglutide	2014	Type 2 diabetes	Nausea, vomiting, diarrhea	Hypersensitivity reaction History of pancreatitis or develops pancreatitis Family history of medullary thyroid cancer or multiple endocrine neoplasia 2 (MEN2)
	2020	Cardiovascular events in adults with/without established cardiovascular disease		
Exenatide	2005	Type 2 diabetes	Nausea, vomiting, diarrhea	Hypersensitivity reaction History of pancreatitis or develops pancreatitis Family history of medullary thyroid cancer or multiple endocrine neoplasia 2 (MEN2) End-stage renal disease or CrCl < 30mL/min Drug induced thrombocytopenia
Tirzepatide	2022	Type 2 diabetes	Nausea, vomiting, diarrhea	Hypersensitivity History of pancreatitis or develops pancreatitis Family history of medullary thyroid cancer or multiple endocrine neoplasia 2 (MEN2)
	2023	Chronic weight management		
	2024	Obstructive sleep apnea		
Lixisenatide *discontinued	2016	Type 2 diabetes	Nausea, vomiting, diarrhea, Dizziness	Hypersensitivity reaction End-stage renal disease or severe renal impairment

In relation to diabetes, CDC estimates show that 38.1 million adults or 14.7% of the US population had diabetes in 2021. Of these, 8.7 million were undiagnosed. The prevalence of diabetes is age-dependent and increases from 4.8% in young adults ages 18–44 to 29% among those older than 65 years. Furthermore, racial and ethnic variations have been observed, with a prevalence of 13.6% in non-Hispanic Whites, 15.5% in Hispanics, and 17.4% in Blacks. Among all patients with diabetes, 80–90% have type 2 diabetes, which is characterized by a reduced secretion of insulin by the beta cells in the pancreas or insulin resistance. Diabetes amplifies the detrimental effect of cardiometabolic risk factors, such as dyslipidemia and atherosclerosis, and is considered a major cause of cardiovascular disease, stroke, and cognitive decline. According to the American Diabetes Association, the total annual cost of diabetes in 2022 was $412.9 billion, accounting for 1 in every 4 healthcare dollars spent [11]. Thus, there is an urgent need for identifying effective treatments for diabetes. By stimulating the endogenous secretion of insulin, reducing the production of glucagon, and delaying gastric emptying, GLP1AR reduce glucose levels and enhance metabolic control. The beneficial effect of GLP1AR for patients with type 2 diabetes has been demonstrated in several RCT. The Liraglutide Effect and Action in Diabetes (LEAD) series were 6 phase 3 RCT that showed improvement in hemoglobin A1c levels, enhanced beta cell function, weight loss, and reduced blood pressure with liraglutide alone or in combination with other oral hypoglycemic agents. Similarly, the Semaglutide Unabated Sustainability in Treatment of Type 2 Diabetes (SUSTAIN) studies was a series of 7 RCT that investigated the efficacy and safety of subcutaneous semaglutide in over 8000 patients with type 2 diabetes [12]. The SUSTAIN 1–5 and 7 trials compared semaglutide to either placebo or other existing hypoglycemic agents. In these studies, semaglutide demonstrated superior and sustained glycemic control and weight loss than all the other comparators evaluated. SUSTAIN 6, in particular, included patients with type 2 diabetes who also had CVD, chronic kidney disease, or both. In this study, patients were randomized to receive subcutaneous semaglutide (0.5 mg or 1.0 mg) once a week or placebo. After 104 weeks of treatment, the primary outcome of cardiovascular death, nonfatal myocardial infarction, or nonfatal stroke occurred in 6.6% of the patients in the semaglutide group and 8.9% of those in the placebo group, resulting in a hazard ratio (HR) of 0.74 (95% CI 0.58–0.95) [13]. The SUSTAIN studies confirmed that, in addition to their effect on glycemic and weight control in patients with type 2 diabetes, GLP1RAs have the potential to reduce the incidence of major adverse cardiovascular outcomes in high-risk populations. In a comprehensive meta-analysis including 76 RCT and more than 39 thousand participants with type 2 diabetes, treatment with GLP1RAs was associated with a reduction in HbA1c of -0.6% to -2.1% as well as reduction of -10.8 to -57.7 mg/dl in the fasting blood glucose levels relative to placebo [14]. Several GLP1RAs have received FDA approval for the treatment of type 2 diabetes, including semaglutide, tirzepatide, liraglutide, dulaglutide, and exenatide (Table 2).

### Effect on Cardiovascular and Cerebrovascular Diseases

The beneficial effect of GLP1RAs on obesity and diabetes and the results of SUSTAIN 6 suggest that this drug class may play a major role in the prevention of CVD and stroke. Thus far, 7 cardiovascular outcomes trials (CVTO) have produced robust data supporting the beneficial effect of GLP1RAs on major adverse cardiovascular events (MACE). In the Liraglutide Effect and Action in Diabetes: Evaluation of Cardiovascular Outcome Results (LEADER) study, patients with type 2 diabetes and high cardiovascular risk were randomized to receive a daily subcutaneous injection liraglutide or placebo. The primary outcome was the first occurrence of MACE, defined as death from cardiovascular causes, nonfatal myocardial infarction, or nonfatal stroke. In the median follow up of 3.8 years, the primary outcome occurred in 13% of the patients randomized to liraglutide and approximately 15% of those receiving placebo (*p* = 0.01 for superiority [15]. Also, in the Researching Cardiovascular Events With a Weekly Incretin in Diabetes (REWIND) study, approximately 10 thousand patients with type 2 diabetes and history of CVD or cardiovascular risk factors were randomized to a weekly subcutaneous injection of dulaglutide 1.5 mg or placebo [16]. The median follow up was 5.4 year, 12.0% of the patients receiving dulaglutide and 13.4% of those assigned to the placebo group experienced the primary composite endpoint of non-fatal myocardial infarction, non-fatal stroke, or death from cardiovascular causes (*p* = 0.026). Most of the GLP1RAs are administered subcutaneously. In an attempt to dispel concerns related to injection site reactions and enhance convenience and patient preference, new oral formulations have been recently developed. The Trial Investigating the Cardiovascular Safety of Oral Semaglutide in Subjects With Type 2 Diabetes (PIONEER 6) randomized patients with high CVD risk to once daily oral semaglutide versus placebo [17]. The primary outcome was a composite of death from cardiovascular causes, nonfatal myocardial infarction, or nonfatal stroke. During the median follow up of 15.9 months, 3.8% of the patients assigned to the study group experienced the primary outcome compared to 4.8% among those in the placebo. Similarly, in the A Heart Disease Study of Semaglutide in Patients With Type 2 Diabetes (SOUL) study, which randomized patients with type 2 diabetes and atherosclerotic cardiovascular disease, chronic kidney disease, or both to oral semaglutide versus placebo, the primary outcome of major adverse cardiovascular event occurred in 12.0% of the patients receiving semaglutide and 13.8% of the patients receiving placebo (hazard ratio, 0.86; 95% confidence interval, 0.77 to 0.96; *P* = 0.006) [18]. Detailed information about these and other trials can be found in Table 3.

**Table 3:** Major cardiovascular outcomes trials

Trial	Primary composite outcome	Trial Specifics	Primary outcome	Take home points	Additional outcomes and possible complications
LEADER (2016) Liraglutide and Cardiovascular Outcomes in Type 2 Diabetes	First occurrence of death from cardiovascular causes, nonfatal MI, or nonfatal stroke	*N* = 9340 Mean HbA1c 8.7 72.4% with CVD 24.7% with CKD stage 3 or greater Median follow up 3.8 years Median daily dose 1.78 mg	Primary outcome occurred in significantly fewer patients (608 of 4668 patients [13.0%]) than in the placebo group (694 of 4672 [14.9%]) (HR = 0.87; 95% CI 0.78 to 0.97; *p* < 0.001 for noninferiority; *p* = 0.01 for superiority)	Frequencies of nonfatal MI or stroke similar in liraglutide and placebo group (HR = 0.89, 95% CI 0.72–1.11; *P* = 0.30)	Pancreatic cancer increased in comparison to placebo, however not significant.
SUSTAIN-6 (2016) Semaglutide and Cardiovascular Outcomes in patients with Type 2 Diabetes	First occurrence of cardiovascular death, nonfatal myocardial infarction, or nonfatal stroke	*N* = 3297 Mean HbA1c 8.7 Medication administration 104 weeks 58% w/ CVD 83% w/ CVD and CKD	Primary outcome occurs in 108 of 1648 patients (6.6%) in semaglutide vs. 146/1649 (8.9%) in placebo group (HR = 0.74; 95% CI 0.58–0.95; *p* < 0.001 for noninferiority and *p* = 0.02 for superiority)	Nonfatal stroke occurred in 1.6% and 2.7%, respectively (HR = 0.61; 95% CI 0.38–0.99; *P* = 0.04)	Lower rate of nephropathy Diabetic retinopathy complications in 50 patients in semaglutide compared to 29 in placebo group. Retinopathy complications including vitreous hemorrhage, blindness or need for photocoagulation significantly higher (hazard ratio, 1.76; 95% CI, 1.11 to 2.78; *P* = 0.02)
REWIND (2019) Dulaglutide and cardiovascular outcome in type 2 diabetes	First occurrence of cardiovascular death, nonfatal myocardial infarction, or nonfatal stroke	*N* = 9901 Median HbA1c 7.2% Median follow up 5.4 years	Primary composite occurred in 594 (12·0%) participants vs. 663 (13.4%) participants in the placebo group (HR = 0.88, 95% CI 0.79–0.99; *p* = 0.026)	*From secondary analysis*, non-fatal stroke was also significantly lower in the dulaglutide group compared with placebo (2.7% vs. 3.5%, *p* = 0.017).	Fewer renal outcomes 17.1% vs. 19.6%, *p* = 0.0004) with no significant difference in retinal complications
HARMONY (2018) Albiglutide and cardiovascular outcomes in patients with type 2 diabetes and cardiovascular disease	First occurrence of cardiovascular death, nonfatal myocardial infarction, or nonfatal stroke	*N* = 9463 Median follow up 1.5 years Mean HbA1c 8.7% 71% w/ prior CVD, 25% peripheral artery disease, and 25% stroke	Primary outcome 338 (7%) of 4731 patients in the albiglutide group and in 428 (9%) of 4732 patients in the placebo group (HR = 0.78, 95% CI 0.68–0.90), (*p* < 0.0001 for noninferiority and *p* = 0.0006 for superiority)	Albiglutide reduced the risk of the primary composite outcome by 22%, compared with placebo	Acute pancreatitis 10 patients compared to 7 in the placebo group, pancreatic cancer in 6 vs. 5 in the placebo group. No statistical significance **Did not require forced uptitration of medication, free to use dpp4 inhibitors and SGLT2 inhibitors
EXSCEL (2017) Effect of Once Weekly Exenatide on cardiovascular Outcomes in Type 2 Diabetes	First occurrence of cardiovascular death, nonfatal myocardial infarction, or nonfatal stroke	*N* = 14,752 Median follow up 3.2 years Median HbA1c 8% 73.1% with prior CVD	Primary outcome 839 of 7356 patients (11.4%) in the exenatide group and in 905 of 7396 patients (12.2%) group (HR = 0.91; 95% CI 0.83-1.00), *P* < 0.001 for noninferiority, *P* = 0.06 for superiority )	Superiority not achieved from primary outcome	Rates of death, fatal/nonfatal stroke, acute pancreatitis, medullary thyroid carcinoma did not differ significantly from both groups
PIONEER-6 (2019) Oral Semaglutide and Cardiovascular Outcomes in Patients with Type 2 Diabetes	First occurrence of major adverse cardiovascular event (death from CVD causes, nonfatal MI, nonfatal stroke)	*N* = 3183 Median follow up 15.9 months Mean HbA1c 8.2%	Primary composite outcome occurred in 3.8% of patients receiving oral semaglutide compared with 4.8% receiving placebo, which was not significant (*p* = 0.17) Nonfatal myocardial infarction, 37 of 1591 patients (2.3%) and 31 of 1592 (1.9%), respectively (hazard ratio, 1.18; 95% CI, 0.73 to 1.90)	First events of nonfatal stroke occurred in 12 of 1591 patients (0.8%) and 16 of 1592 (1.0%), respectively (hazard ratio, 0.74; 95% CI, 0.35 to 1.57). Death from CV cause 15 of 1591 patients (0.9%) in the oral semaglutide and 30 of 1592 (1.9%) in the placebo group (HR = 0.49; 95% CI 0.27–0.92)	Diabetic retinopathy 7.1% (113 of 1591 patients) with oral semaglutide and 6.3% (101 of 1592) with placebo. Severe hypoglycemia was 1.4% (23 of 1591 patients), as compared with 0.8% (13 of 1592) with placebo. Patient also receiving other diabetes medication including insulin or sulfonyl ureas
AMPLITUDE (2021) Cardiovascular and Renal Outcomes with Efpeglenatide in Type 2 Diabetes	First occurrence of major adverse cardiovascular event MACE (nonfatal myocardial infarction, nonfatal stroke, or death from cardiovascular or undetermined causes)	*N* = 4076 Median follow up 1.81 years 89.6% w/ CVD, 31.6% w/ eGFR < 60 ml/min 15.2% using SGLT2i	MACE occurred in 189 participants (7.0%) using efpeglenatide and 125 participants (9.2%) using placebo (HR = 0.73; 95% CI 0.58–0.92; *p* < 0.001 for noninferiority; *P* = 0.007 for superiority)	Lower renal outcome with 53 participants (13.0%) assigned to receive efpeglenatide and in 250 participants (18.4%) assigned to receive placebo (HR = 0.68; 95% CI 0.57–0.79; *P* < 0.001)	Systolic blood pressures twere lower by 1.5 mm Hg (95% CI, 0.8 to 2.2) and diastolic blood pressures 0.6 mm Hg (95% CI, 0.2 to 1.0). Potential protective effect on cardiovascular health and kidneys. *Could take SGLT2i

CI = confidence interval; CKD = chronic kidney disease CVD = cardiovascular disease; HR = hazard ratio; MACE = major adverse cardiovascular events; SGLT2i =sodium-glucose cotransporter-2 inhibitors (

Although stroke was not a primary outcome in RCT that assessed the effect of GPL1RA in high-risk patients, subgroup analyses of several CVOTs suggest that these medications may reduce the risk of cerebrovascular injury. In a secondary analysis of REWIND, the incidence of non-fatal stroke was 2.7% in the dulaglutide group compared with 3.5% in the placebo group (HR = 0.76; 95% CI 0.61 to 0.95; *p* = 0.017) [19]. In a meta-analysis including seven CVOTs and approximately 56 thousand participants, the use of GLP1RAs in patients with type 2 diabetes reduced the risk of total stroke by 16% (HR = 0.84; 95% CI 0.46–0.93) and the risk of non-fatal stroke by 15% (HR = 0.85; 95% CI 0.76–0.94) relative to placebo [20]. In SUSTAIN- 6 nonfatal stroke occurred in 1.6% in the semaglutide group vs. in 2.7% of those receiving placebo, resulting in a reduction of 39% in the hazard ratio in favor of the GLP1AR (HR = 0.61; 95% CI 0.38 to 0.99; *p* = 0.04) [13]. Furthermore, in another meta-analysis including over 30 thousand patients, GLP1RAs reduced the risk of all total stroke by 17% (HR = 0.83; 95% CI 0.76–0.92) and the risk of non-fatal stroke by 16% (HR = 0.84; 95% CI 0.76–0.93), relative to placebo [21].

Different mechanisms may explain the beneficial effect of GLP1RAs on stroke prevention. Low-density lipoproteins are pro-atherogenic and have been linked to intracranial and extracranial atherosclerotic disease. The results of a meta-analysis including 33 studies show that GLP1RAs have a modest, albeit significant, effect on lipid profile which is independent of their effect on weight reduction. Patients receiving GLP1RAs, in particular, showed a reduction of 2.93 mg/dl in LDL-cholesterol levels and 5.52 mg/dl in total cholesterol levels with no conclusive effect in triglycerides or HDL-cholesterol. It is worth noting that this meta-analysis showed a large heterogeneity across studies (I^2^ > 99%), suggesting that there may be differences on the effect of different GLP1RAs on lipid metabolism [22]. Small dense LDL (sdLDL) particles are highly susceptible to oxidation and are more atherogenic than LDL-cholesterol. Data obtained in a small cohort of patients with type 2 diabetes suggest that liraglutide reduces sdLDL levels and carotid intima-medial thickness [23]. A similar effect was described in association with tirzepatide, a GIP-R/GLP-1R co-agonist [24]. In addition, data obtained in preclinical models and small cohorts suggest that the GLP1RAs liraglutide may downregulate PCSK9, a major inhibitor of LDL-receptor expression associated with the development of atherosclerosis [25, 26].

There is emerging evidence that supports the active role of GLP1R signaling pathways in microvascular function and blood pressure control. GLP-1 receptors are expressed in endothelial and vascular smooth muscle cells of conduit and resistance arteries. Results obtained in preclinical models indicate that GLP-1 improves endothelial dysfunction, prevents vascular remodeling, and activates the endothelial nitric oxide synthase-nitric oxide-cGMP pathway. Furthermore, through the activation of canonical G-protein coupled receptors, GLP-1 receptors activate adenylyl cyclase which leads to an increased production of cAMP, a second messenger involved in vasodilation [27]. GLP-1 can also support microvascular health by inhibiting angiotensin II-NADPH oxidase, which reduces the production of reactive oxygen species in endothelial cells. It should be noted that carotid body sensitization participates in sympathetic nerve activity in hypertension. Data obtained in animal models support the idea that GLP-1 reduces sympathoexcitation and induces natriuresis, both processes associated with hypertension. These findings correlate with the results obtained in the REWIND study, where the use of dulaglutide was associated with a reduction of the systolic blood pressure of 1.7 mmHg when compared to placebo [16]. Also, in the SCALE study, liraglutide reduced the systolic blood pressure by 2.8 mmHg compared to placebo and, in the STEP-1 trial, semaglutide reduced the blood pressure by 5.1 mmHg systolic and 2.4 mmHg diastolic compared to placebo. Furthermore, the use of once weekly subcutaneous tirzepatide, a dual GLP1RAs and GIP agonist, reduced the systolic blood pressure from baseline by 7.4 to 10.6 mmHg, compared to placebo [28].

There is emerging data supporting the notion that GLPRA may be beneficial in atrial fibrillation (AF) and heart failure (HF), both important risk factors for stroke. In relation to AF, data obtained in animal models suggest that, by reducing oxidative stress and inflammation, subcutaneous liraglutide can reduce atrial enlargement as well as structural and electrical remodeling, all processes that contribute to the development and perpetuation of AF [29]. In a meta-analysis including 11 RCT, semaglutide was shown to decrease the occurrence of incident AF by almost 42% compared to placebo [30]. In addition, results obtained using the experimental models of HF with preserved ejection fraction showed that semaglutide improves endothelial function and left ventricular cytoskeleton function, and restores the cardiometabolic milieu. This was associated with an improvement in cardiac structure and function [31]. These findings correlate with the results obtained in a meta-analysis of 6 CVOT that evaluated GLP1RAs in HF with preserved ejection fraction. In this study, GLP1RAs reduced the composite of CV death and worsening HF by 32% (HR = 0.68; 95% CI 0.51–0.89) and worsening HF alone by 44% (HR = 0.56; 95% CI 0.38–0.82) [32].

Lastly, obesity is a risk factor for venous thromboembolism (VTE). In preclinical models, GLP1RAs inhibit platelet aggregation, reduce platelet activation markers, and suppress thromboxane A2 production [33]. These findings suggest that these drugs may prevent VTE. In a target trial emulation study, GLP1RA reduced the incidence of VTE by 22% (HR = 0.78; 95% CI 0.73–0.8) [34]. However, the evidence obtained in RCTs, albeit confounded by a low incidence of VTE events, has yielded inconclusive results [35].

Based on the available information, the 2024 AHA Guideline for the Primary Prevention of Stroke recommends using GLP1RAs in patients with diabetes and high cardiovascular risk or established CVD and hemoglobin A1c ≥ 7% (Class of Recommendation 1, Level of Evidence A) [36].

### Effect on Cognition

Improvements in medical care have contributed to a global rise in life expectancy. This progress has been linked to a significant increase in the incidence and prevalence of age-related diseases, such as cognitive impairment and dementia. GLP1RAs can be effective for the treatment of obstructive sleep apnea, a condition associated with elevated white matter hyperintensity burden, microstructural damage, and cognitive impairment [37]. In particular, the treatment of adults carrying the diagnosis of obesity and moderate-to-severe obstructive sleep apnea with tirzepatide for 52 weeks has been associated with reduced levels of markers of systemic inflammation and improved sleep-related outcomes, including the apnea-hypopnea index, body weight, hypoxic burden, systolic blood pressure, and sleep-related patient-reported outcomes [38]. In addition, vascular risk factors, stroke, and CVD contribute to both vascular and neurodegenerative dementias [6, 39, 40]. Thus, it is plausible that GLP1RAs could reduce the burden of cognitive impairment and mental health disorders in the general population [41].

In a network meta-analysis, the odds of dementia among individuals treated with dulaglutide, exenatide, or liraglutide was reduced by 66% relative to non-GLP1AR users [42]. In addition, in a meta-analysis that included 26 RCT comparing cardioprotective glucose-lowering therapy with controls that reported dementia or change in cognitive scores, glucose-lowering therapy was not associated with a reduction in cognitive impairment or dementia. However, when analyzed by drug class, GLP1RAs almost halved the odds of all-cause dementia (OR = 0.55; 95% CI, 0.35–0.86), but sodium-glucose cotransporter-2 inhibitors (SGLT2i) did not modify it (OR = 1.20, 95% CI, 0.67–2.17) [43]. The interpretation of these findings is confounded by the low number of dementia cases in each study. Nevertheless, these results align well with data from the OneFlorida + Data Trust healthcare repository. In this study, the authors compared the risk of Alzheimer’s Disease and Related Dementias (ADRD) among approximately 50 thousand patients receiving GLP1RAs, SGLT2i, or other glucose-lowering drugs. During the mean follow-up of approximately 2 years, there were 75 ADRD cases among GLP1RAs users, 639 cases among GLD users, and 101 cases among SGLT2i users. The calculated risk of ADRD was lower among GLP1RAs users than in those treated with other glucose-lowering drugs (HR = 0.67; 95% CI, 0.47–0.96), but comparable among patients treated with GLP1RAs and SGLT2i (HR = 0.97; 95% CI, 0.72–1.32) [44]. Adequately powered studies with prespecified cognitive outcomes and long follow-up are necessary to ascertain the effect of GLP1RAs on age-related cognitive change. Currently, the randomized studies EVOKE (NCT04777396) and EVOKE Plus (NCT04777409) are studying the efficacy of oral semaglutide on the progression of cognitive decline in patients with early Alzheimer’s disease.

### Side Effects and Other Concerns

GLP1RAs are well tolerated and, as a group, the most common adverse events resulting in the discontinuation of treatment are gastrointestinal complications, including nausea, vomiting, diarrhea, and abdominal discomfort. Albeit infrequent, severe gastrointestinal complications, such as pancreatitis and bowel obstruction, have also been described. In SUSTAIN-6, retinopathy and vitreous hemorrhage requiring photocoagulation were seen more commonly in patients receiving high-dose semaglutide than in those assigned to the placebo arm [13]. Additionally, most of the trials excluded patients with family history or personal history of multiple endocrine neoplasia type 2.

The rapid increase in the use of these medications has led to supply challenges and resulted in the proliferation of compounded analogs, raising concerns about safety and bioequivalence [45]. Additionally, the high cost of these therapies has driven up pharmaceutical expenditures and sparked concerns about equitable access, especially among minority populations, who face a disproportionately higher burden of CVD and metabolic syndrome. One significant concern is the potential for treatment discontinuation due to cost, drug shortage, or adverse effects, which can lead to a deterioration in key cardiometabolic parameters, including glycemic control, blood pressure, and cholesterol levels. Moreover, it has been suggested that lean muscle mass lost during treatment with GLP1RAs may not be fully recovered after discontinuation, potentially resulting in a return to pretreatment cardiometabolic risk or a rebound risk that exceeds baseline levels [46].

## Conclusion

GLP1RAs represent a novel class of therapies that significantly improves cardiometabolic profiles and reduces the risk of CVD, stroke, and potentially neurodegenerative conditions. Current practice guidelines recommend their use in populations with high cardiovascular risk. To maximize therapeutic benefit, healthcare providers should closely monitor treatment adherence and ensure patients are well informed about the potential rebound in cardiometabolic risk following treatment discontinuation.

## Key References

- McGuire DK, Busui RP, Deanfield J, Inzucchi SE, Mann JFE, Marx N, et al. Effects of oral semaglutide on cardiovascular outcomes in individuals with type 2 diabetes and established atherosclerotic cardiovascular disease and/or chronic kidney disease: Design and baseline characteristics of SOUL, a randomized trial. Diabetes Obes Metab. 2023;25(7):1932-41. 10.1111/dom.15058.
-
  - Large double-blind, placebo controlled trial comparing once daily oral semagluide and placebo in additional to standard of care. The outcomes included MACE as well as major kidney disease.
- Testai FD, Gorelick PB, Chuang P-Y, Dai X, Furie KL, Gottesman RF, et al. Cardiac Contributions to Brain Health: A Scientific Statement From the American Heart Association. Stroke. 2024;55(12):e425-e38. 10.1161/STR.0000000000000476.
-
  - Comprehensive review article discussing heart failure, atrial fibrillation, and coronary heart disease linking them to brain health including dementia and stroke.
- Zheng ZZ, Yao; Ma, Yiyang. Glucagon-like peptide-1 receptor: mechanisms and advances in therapy. Signal Transduction and Targeted Therapy. 2024;234(9). 10.1038/s41392-024-01931-z.
-
  - Comprehensive review article discussing recent clinical trials with GLP1RA with insight on neuroprotection and inflammation.

## Data Availability

No datasets were generated or analysed during the current study.
